# Reproductive cancer risk factors among relatives of cancer patients in a tertiary oncology center

**DOI:** 10.1186/s12885-019-5350-9

**Published:** 2019-02-15

**Authors:** Nooria Atta, Saadettin Kilickap, Deniz Yuce, Mutlu Hayran

**Affiliations:** 1 Department of Obstetrics and Gynecology, Kabul Medical University, Kabul, Afghanistan; 2 Department of preventive Oncology, Hacettepe University Cancer Institute, Ankara, Turkey

**Keywords:** Reproductive cancer, Risk factor, Early detection of cancer, Cancer screening

## Abstract

**Background:**

The aim of this study was to evaluate the prevalence of some gynecological cancer risk factors in a population of female relatives of cancer patients in Hacettepe University Oncology Hospital. Additionally, what are the levels of the women’s awareness /behavior toward available screening tools?

**Methods:**

An individual cancer risk assessment questionnaire has been developed in the Department of Preventive Oncology, which questions the medical history, health behaviors and cancer awareness, as well as their behavior toward available cancer screening tools.

**Results:**

The mean age of the study population was 45.7 ± 12.2 years. Median age at menarche was 13 years (IQR, 12–14), 6.9% of the women reported their menarche was before age of 12. About 11.1% of the women had intercourse before age of 18. The median age at first delivery was 22 years. Median BMI was 24.9, with 18.3% of population having obesity. Of the women 65% were current/past smokers. Sixty-two percent of the women had never used condom. About 8% of the women were unaware about mammography and 17.7% about the Pap test.

**Conclusions:**

This study has documented high tobacco use, low protective condom use and low rates of physical activity. Percentage of some risk factors like early menarche was lower than what was suggested for general Turkish population. Awareness and behavior of the women were better about mammography when compared to the Pap test. Considering our results, some measures should be put in place to increase people’s awareness, and to modify their behavior toward cancer prevention.

## Background

Cancer by causing 8.2 million deaths in 2012 is one of the leading causes of death worldwide. Globally, there were 14.1 million new cases of cancer [1]. The number of cancer cases will reach 24 million a year by 2035, but half could be prevented. Gynecological cancers, which primarily originate from ovaries, uterine corpus, uterine cervix, vulva and vaginal tissues, represent more than 16% of all cancers. Its age standardized incidence rate is about 29/100,000/year. In Turkey, the rate of gynecologic cancers is 21.3 [1].

Overall etiology of gynecologic cancers is not well understood. However, some of risk factors are known for these cancers. Some of the risks are modifiable like obesity, hormone therapy, age at first sex, age at first birth, number of sex partners. While, some others are not for example age, familial history of cancers [2]. On the other hand some factors have an increasing effect to almost all gynecologic cancers such as: age, hormone therapy, obesity, and others [3, 4]. While some others may have different effect; such as parity, which increases the risk of cervical cancer but decreases the risk of endometrial cancer [5, 6]. The distribution of risk factors for gynecologic cancers as well as other cancers is not same in all parts of the world [3]. Assessing cancer risk in average and high-risk people is essential for primary prevention and early detection of cancers in women. The evidences indicate that in some cancers by taking preventive measures, screening and early detection almost all cases can be prevented primarily and secondly [6, 7]. In Turkey, to our knowledge, there has not been done a risk assessment study yet. To do risk assessment, defining the risk factors’ prevalence in a particular population is vital.

The aim of this study is to evaluate the prevalence of some reproductive cancer risk factors in a population of female relatives of cancer patients. Additionally, evaluate the women’s awareness /behavior toward available screening tools.

## Methods

This is a cross-sectional study, which has been done in Hacettepe University Cancer Institute’s Preventive Oncology Department. A cancer risk assessment questionnaire had been designed by the Department of Preventive Oncology and filled by relatives of cancer patients coming to the Hacettepe University Oncology Hospital in order to be diagnosed and/or treated. Answering to the questionnaire was voluntary. The questionnaire was completely self-administrated.

In this study, we primarily measured frequencies of specific risk factors for gynecologic cancers. In addition, level of women’s awareness of available screening tools and their behavior toward them has been measured.

SPSS 22.0 package was used for data analysis. To analyze data, first descriptive statistical methods have been applied. To compare groups of data; for normal distributed two independent groups, the independent samples t-test was conducted and ANOVA in case of more than two groups. The Tukey test was used for subgroup analysis. For non normally distributed data, Mann- Whitney-U for two groups or Kruskall-Wallis tests for more than tow group were conducted. Chi-square and Fisher’s tests were used to determine the association between categorical data. To test the significance of the pairwise differences Bonferroni correction was used to adjust for multiple comparisons. The significance level was set at *p* < 0.05.

## Results

This study included a total of (*n* = 595) women with a mean (SD) age of 45.7 (12.2) years (ranged from 15 to 86). The majority (68%) of the women were currently married (Table 1).

**Table 1:** Summary descriptive values

Variable (valid data)	Group	Mean (SD)	N (%)
Age, yrs. (*N* = 509) Mean ± SD (45.7 ± 12.2)	≤ 35	29.4 (4.4)	121 (23.8)
	36–55	46.2 (5.7)	276 (54.2)
	≥ 56	62 (5.2)	112 (22)
Marital status (*N* = 558)			
	Married		379 (67.9)
	Single		119 (21.3)
	Divorced		37 (6.6)
	Widow		23 (4.1)
Education (*N* = 570)			
	≤ Intermediate		240 (35.7)
	High school		185 (32.5)
	≥ University		181 (31.8)
Age at menarche (*N* = 478) Median (IQR); 13 (12–14)	Age < 12		33 (6.9)
	Age 12–15		402 (84.1)
	Age > 16		43 (9)
Menstruation (*N* = 518)			
	No		153 (29.5)
	Yes, regular		267 (51.6)
	Yes, irregular		98 (18.9)
Pregnancy (*N* = 516)			
	Yes		392 (76)
	No		124 (24)
Age at first delivery (*N* = 336) median (IQR), 22 (19–26)	< 18		31 (9.2)
	18–29		265 (78.9)
	> 29		40 (11.9)
Number of Delivery (*n* = 334) Median (IQR); 2 (2–3)			
	≤ 2		230 (68.9)
	3 --5		95 (28.4)
	≥ 6		9 (2.7)
Breast feeding (*N* = 502)			
	Yes		354 (70.5)
	No	–	148 (29.5)
Age at first intercourse (*N* = 322) median (IQR), 21 (18–24)			
	≥18		264 (82)
	< 18		58 (18)

Median menarche age of the women in this study was 13 (IQR, 12–14). Only 6.9% of them reported menarche before age 12). Moreover, 18.9% of the women reported irregular menstrual bleeding.

Median age of first sexual activity was 21 (IQR, 18 to 24), and 18% reported early sexual activity (at age < 18 year).

The majority (76%) of participants had been at least once pregnant in their lifetimes. The median age of the first delivery in this study was 22 (IQR 19 to 26), and the median number of deliveries in our study population was 2 (IQR, 2 to 3). Only 9.2% of the women reported their first delivery age younger than 18 years.

Median BMI of our study population was 24.9. Of them 18.3% were obese and 31.1% were overweight.

Seven percent of them were cancer survivors, while proportion of Hypertensive and Diabetic patients were 14.3 and 6.2% respectively.

Majority (64.6%) of the women reported themselves as current or past cigarette smokers.

Answering about their sport habits, about half (49.2%) of the women, who answered this question said they never do sport.

About 11% of the women reported more than one sexual partner. Considering the available data about condom usage, 62.1% of them had never used it. About 8.1% of the women had no awareness about mammography. Seventeen percent of the women participating in this study had no awareness about Pap smear (Table 2).

**Table 2:** Summary descriptive values of personal/familial medical and behavior/ awareness variables

Variable (valid data)	Group	Median (IQR)	N (%)
BMI (*N* = 453) median (IQR) 24.9 (21,7–28.2))	Age ≤ 35	21.5 (20–24.2)	110 (24.3)
	Age = 36–55	25.4 (22.2–28.7)	234 (51.7)
	Age ≥ 56	26.9 (24–30)	88 (19.4)
Interpretation of BMI (N = 453)	Normal (BMI ≤ 25)		229 (50.6)
	Overweight (25.1–30)		141 (31.1)
	Obese (BMI ≥ 30.1)		83 (18.3)
History of chronic diseases	Cancer		40 (7)
	Hypertension		85 (14.3)
	Osteoporosis		47 (8)
	Diabetes		37 (6.2)
	Colorectal adenoma		7 (1.2)
Family history of cancer (*N* = 551)			
	Yes		363 (65.9)
	No	–	188 (34.1)
Oral Contraceptive Pills (*N* = 487)			
	Never		359 (73.7)
	Yes, discontinued	2 yrs. (1–4.5)	98 (20.1)
	Yes, currently	3 yrs. (1–5.5)	30 (6.2)
Tamoxifen (*N* = 433)			
	Never		421 (97.2)
	Yes, discontinued	5 yrs. (3.5–5)	5 (1.2)
	Yes, currently	3 yrs. (1–3)	7 (1.6)
H/O STDs (*N* = 470)			
	No		458 (97.4)
	Yes		12 (2.5)
Smoking (*N* = 495)			
	Yes, now/past		320 (64.6)
	No		175 (35.4)
Number of sex partners (*N* = 298)			
	One		265 (88.9)
	2--4		27 (9.1)
	> 4		6 (2)
Condom use (*N* = 377)			
	Never		234 (62.1)
	Sometimes		79 (21)
	Often		30 (8)
	Always		34 (9)
H/O Mammography (*N* = 480)			
	No awareness		39 (8.1)
	No, don’t want to do		159 (33.1)
	No, going to do		89 (18.5)
	Yes, will not repeat		91 (19)
	Yes, will repeat		102 (21.3)
H/O Pap test (*N* = 479)			
	No awareness		82 (17.1)
	No, don’t want to do		133 (27.8)
	No, going to do		49 (10.2)
	Yes, will not repeat		106 (22.1)
	Yes, will repeat		109 (22.8)

To compare the age of menarche in different age groups, median age at menarche did not vary significantly among the groups of women participating in this study.

After comparing BMI of women having regular menstruation with women of the irregular menstruating group, it was found that, women with irregular menstruation were more likely to have a higher BMI (*p* < 0.001). Some other variations in BMI according other variable are given in Table 3.

**Table 3:** BMI variations according other variable

	N	BMI - N (%)			
		< 25	25.1–30	> 30.1	*P*- value
Chronic Diseases					
- Diabetes	27	1 (3.7)	11 (40.7)	15 (55.6)	*P* < 0.001
- Hypertension	69	15 (21.7)	21 (30.4)	33 (47.8)	*P* < 0.001
- Osteoporosis	39	18 (49)	10(25.6)	11 (28.2)	*P* = 0.202
Menstruation					
- Regular	240	129 (63)	55 (27)	20 (10)	*P* < 0.001
- Irregular	74	36 (48.6)	23 (31.1)	15 (20.3)	
Marital status					
- Single	90	71 (78.9)	14 (15.6)	5 (5.6)	*P* < 0.001
- Married	297	131 (44)	107 (36)	59 (19.9)	
- Divorced	32	14 (43.8)	9 (28.1)	9 (28.1)	
- Widow		7 (36.8)	5 (26.4)	7 (36.8)	
HRT					
- No	134	50 (37.3)	48 (35.8)	36 (26.9)	*P* = 0.522
- Yes	31	15 (48.4)	6 (19.4)	10 (32.2)	
OCP					
- No	275	139 (50)	83 (30.7)	53 (19.3)	*P* = 0.623
- Yes	105	57 (54.3)	29 (27.6)	19 (18.1)	
Cancer in family					
- No	150	83 (55.3)	42 (28)	25 (16.7)	*P* = 0.128
- Yes	76	130 (47)	91 (33)	55 (19.9)	
Smoking					
- No	129	58 (45.5)	38 (30.2)	32 (24.3)	*P* = 0.035
- Yes	255	140 (55)	78 (30.6)	37 (14.5)	
Monthly income					
- < 500	25	10 (40)	11 (44)	4 (16)	*P* = 0.019
- 500–1000	127	52 (40.9)	45 (35.4)	30 (23.6)	
- 1000–3000	221	117 (53)	64 (29)	40 (18.1)	
- > 3000	44	31 (70.5)	10 (22.7)	3 (6.8)	

## Discussion

This study is an attempt to measure the frequency of specific gynecological cancers’ risk factors in female relatives of cancer patients.

The mean (SD) age at interview of the participants was 45.7 (12.2) years and about 76% of the women were older than 35. In comparison to the general Turkish population - female median age of 29.6- the women participating in this study were older [1, 8]. The proportion of current/past smokers in this study was higher than given proportion (overall 31.1%; female 15.2%) for Turkish general population [1]. There could be some explanations for this difference, such as; higher median age, higher urbanization, and higher levels of education in our study population. However, as smoking is a modifiable cancer risk factor, to prevent cancers, the population should be motivated not to smoke, especially if they have some unmodifiable cancer risks e.g. family history of cancer.

Median menarche age was 13 years and it was higher than what Chumlea WC et al. found for U.S white girls whose median age at menarche was 12.6 years [9]. Median age at menarche in the current study, however, confirms findings of Ekerbicer H C et al., in their study about age at menarche in adolescents in the Eastern Mediterranean city of Kahramanmaras, Turkey, who recorded age at menarche as 13 years [10].

In our study only 6.9% had early menarche (< 12 years). For American girls this percentage was more than 10%. In 2009 Talma et al. reported that, 33% of Turkish girls living in Netherland had early menarche [11].

About 11% of participants reported sexual activity before age of 18. In 2005 Dagdeviren N et al. studied the sexual activity behavior among Turkish adolescent of median age of study population, 18 yrs., and found that, the median age of sexual activity in girls was 17 years, near to western countries [12]. The age distribution of our study population (median, 46 yrs.) was totally different from above mentioned study population, which can explain the difference in sexual behavior. Also, there is a possibility of changing sexual behavior over a period of time. That indicates need of further investigations for evaluating any changes of sexual behavior in the Turkish general population.

In this study median age at first delivery was 22 and the proportion of early delivery was 9.2%, which is higher than what is found in Europe (1% in Germany, 2% in France), consistent with the United States (10%) and lower than in Mali (45%), in Uganda (42%), and 25% for Ethiopia [13].

Sixty-two percent of women in this study, said that they never use the condom, while, 9% always used it. Women with lower education levels had a lower rate of condom usage.

Women in this study had a median BMI of 24.9. Women in the obese group were significantly older compared to those with normal weight. We found that, obesity was more prevalent in nonsmokers when compared to those who smoke. But, women with higher education at the level of university or more and higher income -more than 3000 Turkish Liras (TL)- had lower obesity percentages compared to the others. In addition a higher percentage of women with irregular menstrual bleeding were obese and overweight compared normal menstruating women, the finding was statistically significant (Table 3).

About half of the women reported that, they never do sport. Compared to older women, those with younger age reported exercising less frequently. Considering our results, some measures should be put in place to educate people about exercise and healthy lifestyle.

About 7% (*n* = 40) of the women had a positive past cancer history; breast cancer was the most prevalent.

All participants in this study came to the hospital with their relatives, who were cancer patients, but some of the cancer patients had no genetic relationship with the women i.e. husband and wife. As a result all of the women had not positive family history of cancer. About 66% of them had at least one family history of cancer in first degree relatives. Breast cancer had the highest (18.4%) proportion. Second most common cancer was lung cancer, colorectal cancer (9.8%) was the third, and gynecological cancers (4.6%) were the fourth most common type in their families.

About 8.1% of participants in this study were not aware of mammography; awareness about the Pap test was lower than mammography, 17.1% of the women had no awareness about Pap tests. Considering data about both available screening tools, we can say that the population should be made more aware of these tests.

This study had a nonrandom sampling design and was done on a population with a specific condition, the results could have some variations from what is valid for the general population. However, findings from this study will be valid for similar population groups, such as; relatives of cancer patients.

Considering our findings, there were a variety of reproductive cancer risk factors in female relatives of cancer patients. Some of them like aging, a family history of cancer, could not be prevented or modified while, some others could be modified to prevent cancer, such as; smoking, breast feeding, condom use, and exercise. As a measure of cancer prevention, the population’s awareness about possible ways of the prevention should be increased. By increasing population’s awareness, their behavior toward modifiable risks would mostly be changed. Finally, data from this study and similar studies can be used in developing cancer risk assessment tools, and strategies for cancer prevention.

## Conclusion

Compared to the general population of Turkey, women participating in this study were more likely to be older. Percentage of some risk factors such as; early menarche, early sexual activity, and early child birth was lower than what was estimated in some other populations. While, the prevalence of others like obesity or some chronic disease were almost adherent to what is suggested for turkey population. However, this study has also documented high tobacco use and low protective condom use.

In addition, our findings support required efforts to increase general population awareness about available cancer screening tools. Also, there is a need for motivating women to use available screening tools according to their given protocols.

By modifying risk factors, many of the cancers can be prevented. The results of this study appear the requirement of smoking behavior, lifestyle and exercise modifications for cancer prevention.

The study provides information that will be useful for developing more specific and precise data collection tools specially for measuring risk factors of women’s cancers.

## Abbreviations

BMI: Body mass index
CI: Confidence interval
CIN: Cervical intraepithelial neoplasia
DES: Diethylstilbestrol
HPV: Human papilloma virus
HRT: Hormone replacement therapy
OCP: Oral contraceptive pills
OR: Odds ratio
RR: Relative risk
SCC: Squamous cell carcinoma
SERM: Selective estrogenic receptor modulators
STD: Sexually transmitted disease
STI: Sexually transmitted Infections
